# Diagnosis of skull-base invasion by nasopharyngeal tumors on CT with a deep-learning approach

**DOI:** 10.1007/s11604-023-01527-7

**Published:** 2024-01-27

**Authors:** Junichi Nakagawa, Noriyuki Fujima, Kenji Hirata, Taisuke Harada, Naoto Wakabayashi, Yuki Takano, Akihiro Homma, Satoshi Kano, Kazuyuki Minowa, Kohsuke Kudo

**Affiliations:** 1 Department of Diagnostic Imaging, Graduate School of Medicine, Hokkaido University, N15 W7, Kita-Ku, Sapporo, Hokkaido 060-8638 Japan; 2 Department of Diagnostic and Interventional Radiology, Hokkaido University Hospital, N14 W5, Kita-Ku, Sapporo, Hokkaido 060-8648 Japan; 3 Global Center for Biomedical Science and Engineering, Faculty of Medicine, Hokkaido University, N14 W5, Kita-Ku, Sapporo, Hokkaido 060-8638 Japan; 4 Department of Nuclear Medicine, Hokkaido University Hospital, N14 W5, Kita-Ku, Sapporo, Hokkaido 060-8648 Japan; 5 Medical AI Research and Development Center, Hokkaido University Hospital, N14 W5, Kita-Ku, Sapporo, Hokkaido 060-8648 Japan; 6 Department of Otolaryngology-Head and Neck Surgery, Faculty of Medicine and Graduate School of Medicine, Hokkaido University, N15 W7, Kita Ku, Sapporo, 060-8638 Japan; 7 Faculty of Dental Medicine Department of Radiology, Hokkaido University, N13 W7, Kita-Ku, Sapporo, Hokkaido, 060-8586 Japan

**Keywords:** Head and neck, Nasopharyngeal tumor, Skull-base invasion, Deep learning, Convolutional neural network

## Abstract

**Purpose:**

To develop a convolutional neural network (CNN) model to diagnose skull-base invasion by nasopharyngeal malignancies in CT images and evaluate the model’s diagnostic performance.

**Materials and methods:**

We divided 100 malignant nasopharyngeal tumor lesions into a training (*n* = 70) and a test (*n* = 30) dataset. Two head/neck radiologists reviewed CT and MRI images and determined the positive/negative skull-base invasion status of each case (training dataset: 29 invasion-positive and 41 invasion-negative; test dataset: 13 invasion-positive and 17 invasion-negative). Preprocessing involved extracting continuous slices of the nasopharynx and clivus. The preprocessed training dataset was used for transfer learning with Residual Neural Networks 50 to create a diagnostic CNN model, which was then tested on the preprocessed test dataset to determine the invasion status and model performance. Original CT images from the test dataset were reviewed by a radiologist with extensive head/neck imaging experience (senior reader: SR) and another less-experienced radiologist (junior reader: JR). Gradient-weighted class activation maps (Grad-CAMs) were created to visualize the explainability of the invasion status classification.

**Results:**

The CNN model’s diagnostic accuracy was 0.973, significantly higher than those of the two radiologists (SR: 0.838; JR: 0.595). Receiver operating characteristic curve analysis gave an area under the curve of 0.953 for the CNN model (versus 0.832 and 0.617 for SR and JR; both *p* < 0.05). The Grad-CAMs suggested that the invasion-negative cases were present predominantly in bone marrow, while the invasion-positive cases exhibited osteosclerosis and nasopharyngeal masses.

**Conclusions:**

This CNN technique would be useful for CT-based diagnosis of skull-base invasion by nasopharyngeal malignancies.

## Introduction

Since nasopharyngeal tumors can be detected and diagnosed by a biopsy performed with endoscopy, the foremost role of X-ray computed tomography (CT) or magnetic resonance imaging (MRI) in the management of patients with nasopharyngeal tumors is to determine the extent of the primary tumor and the presence of metastasis. Because the nasopharynx is located close to and below the base of the skull, nasopharyngeal tumors often involve the skull base. Invasion of the skull base is currently classified as T3 according to the 8th edition of the Union for International Cancer Control clinical TNM staging criteria [1] and is considered to be a prognostic factor indicating a high risk of local recurrence and a poor survival rate [2].

Computed tomography reveals permeative or erosive bone changes of the skull base or spread along foraminal pathways. Sclerosis of the pterygoid process with increased attenuation of the medullary cavity or thickening of cortical bone may also be detected by CT [3]. MRI reveals (i) the replacement of high-signal bone marrow with low signal intensity and (ii) the enhancement with gadolinium-based contrast agent due to tumor invasion on T1-weighted imaging (T1WI) [4]. The observance of a high signal on fat-suppressed T2-weighted imaging (FsT2WI) is also helpful in the diagnosis of bone marrow involvement. Compared to nasopharyngeal carcinomas, malignant lymphoma of the nasopharynx tends to have less frequent and less extensive deep invasion [5], but the finding of invasion itself is similar to that of nasopharyngeal carcinoma.

It is generally accepted that CT is superior to MRI in demonstrating bony erosion, whereas MRI is better in delineating soft tissue abnormalities [6]. Several reports have indicated that MRI is more sensitive than CT for detecting skull-base invasion [6–8]; however, another research group reported that erosion of the skull base suggested by only MRI was not always associated with a high risk of local recurrence [9]. It is thus important that clinicians obtain both CT and MR findings in cases of suspected skull-base invasion by a nasopharyngeal tumor.

Moreover, MRI requires a long imaging time, and poor image quality due to motion artifacts may occur, especially in patients with symptoms such as pain or dyspnea. Some individuals are unable to undergo MRI due to claustrophobia, a metal implant, or an electronic device such as a pacemaker. There are thus situations in which it is necessary to evaluate the presence or absence of skull-base invasion using CT.

In various clinical contexts, the application of deep learning (DL) algorithms, particularly with a convolutional neural network (CNN), to medical imaging has recently gained notable interest [10, 11]. Studies have investigated DL techniques for the diagnosis of local invasion of malignant tumors on CT images at various anatomical sites [12, 13], including invasion by head and neck malignancies [14, 15]. The DL approaches were observed to provide clinically sufficient diagnostic performance equivalent to that of a radiologist. In line with these reports, our speculation was that the use of a CNN might enable the determination of skull-base invasion on CT and assist radiologists in the diagnosis of such cases. The present study was conducted to create a CNN model for diagnosing skull-base invasion by a malignant nasopharyngeal tumor on CT images, and then to evaluate diagnostic performance of the model.

## Methods

This retrospective study was approved by the Institutional Review Board of the Hokkaido University, and the requirement for patients’ written informed consent was waived.

### Study population

We selected the cases of 115 patients from the medical records of our hospital between January 2008 and March 2022, based on the following inclusion criteria: patients with (1) a pathologically confirmed malignant nasopharyngeal tumor, (2) pre-treatment axial CT images reconstructed with a soft tissue kernel including the tumor lesion. We excluded some patients because of the following exclusion criteria: (1) CT image slice thickness > 4 mm (*n* = 11), or (2) MRI had not been conducted (*n* = 4). Ultimately, 100 cases were deemed eligible for this study.

### CT and MRI images

Computed tomography images of the 100 cases were obtained by various scanners from four vendors. Of the 100 cases, seven had non-contrast enhanced images only, 76 had contrast-enhanced images only, and the other 17 cases had both non-contrast-enhanced and contrast-enhanced images. Image parameters were as follows. Slice orientation: axial, Slice thickness: 1.25–4.00 mm, matrix size: approximately 512 × 512, reconstruction kernel: soft tissue. MRI images were also obtained by various scanners from five vendors. MRIs were scanned within 2 months before and after patient’s CT imaging, and at least two sequences of T1WI, FsT2WI, gadolinium-enhanced T1-weighted imaging (GdT1WI), and/or fat-suppressed gadolinium-enhanced T1-weighted imaging (FsGdT1WI). Of the 100 cases, 6 had non-contrast enhanced images only, and the other 94 had both non-contrast-enhanced and contrast-enhanced images. The imaging orientation had axial sections and at least one of the sagittal or coronal sections. Slice thickness: approximately 3–5 mm, matrix size: approximately 512 × 512.

### Ground truth determination for skull-base invasion

Two board-certified radiologists with 7 years and 16 years of experience in head and neck radiology assessed the suitability of the CT and MRI images to interpret, using a Digital Imaging and Communication in Medicine (DICOM) viewer (XTREK, J-MAC System, Tokyo, Japan). The range of evaluation on the axial sections was set from the base of the sphenoid sinus to the lower end of the clivus.

For the determination of the ground truth, the two radiologists evaluated the skull-base invasion by consensus based on the presence or absence of MRI findings (low-intensity on T1WI, high-intensity on FsT2WI, and enhancement in bone marrow), CT imaging findings (permeative or erosive bone changes, and/or sclerosis of bone marrow), and the patients’ all-available medical records. In CT image assessment, we essentially used axial sections with appropriate adjustment of the window level/width for the evaluation. Coronal and/or sagittal reconstructed images were also used for the evaluation if available. After this image assessment, 42 cases were diagnosed as invasion-positive and the remaining 58 cases were diagnosed as invasion-negative. Thereafter, for cases classified as invasion-positive, the presence or absence of invasion findings was also assessed for each slice of all CT images within the evaluation range; the details are provided below in the *Image preprocessing and deep-learning analysis* section.

### Configuration of datasets for training and test

**Fig. 1:**
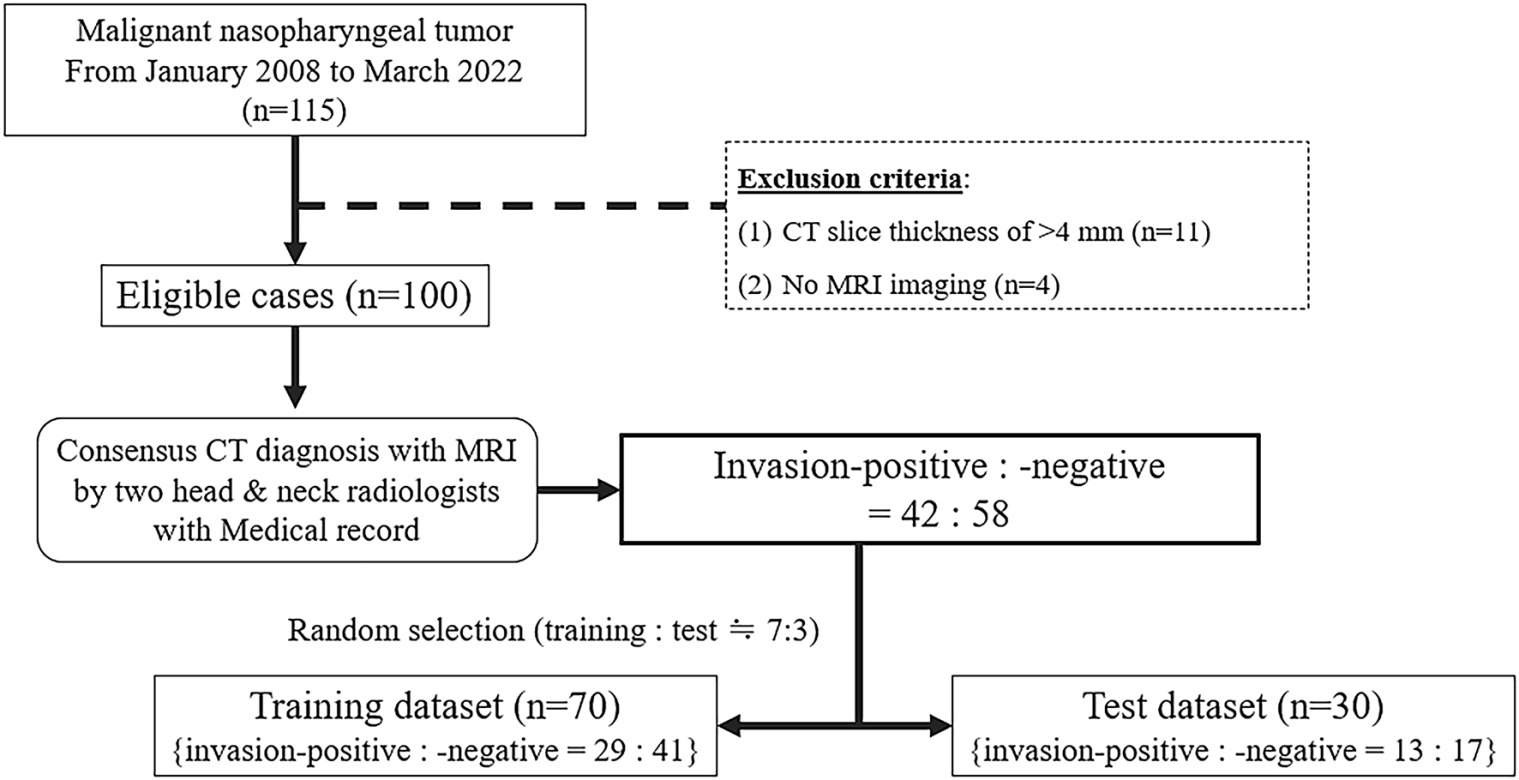
Study population, recruitment pathway, and ground truth labeling

We randomly selected 70 of the 100 cases as the training dataset to establish the diagnostic model, and used the remaining 30 cases as the test dataset to estimate the performance of the established model, so that the datasets’ respective ratios of invasion-positive to invasion-negative cases were maintained. As a result, 29 invasion-positive and 41 invasion-negative cases comprised the training dataset, and 13 invasion-positive and 17 invasion-negatives comprised the test dataset (Fig. 1).

### Image analyses

#### Image preprocessing and deep-learning analysis

First, all axial CT images were segmented, with rectangular regions of interest (ROI) manually drawn to include the nasopharynx and skull base (especially the clivus) with an ROI size of approximately 18 cm^2^. We used the CT images in the vertical range from the base of the sphenoid sinus to the lower end of the clivus, and we set the rectangular ROI range from the pterygoid to the posterior end of the clivus in the anterior–posterior direction and the bilateral internal carotid arteries in the lateral direction. The CT window of all images was set to the window level 60 Hounsfield units (HU) and window widths 600 HU. Finally, all preprocessed images were saved in Joint Photographic Experts Group (JPEG) format.

To create the invasion-positive group in the training dataset, only the specific slices classified as invasion-positive (see the Sect. ‘Ground truth determination for skull-base invasion’) were included. This dataset consisted of 146 images from 33 cases (non-contrast only: *n* = 1, contrast only: *n* = 24, both non-contrast and contrast: *n* = 4). We included all of the extracted slices in the invasion-negative group; this dataset consisted of 226 images from 47 cases (non-contrast only: *n* = 4, contrast only: *n* = 31, both non-contrast and contrast: *n* = 6).

Prior to training, a total of 21 additional images were generated by data augmentation to improve the robustness of the model, by horizontal flipping, random rotating, and vertically and/or horizontally shifting each image. Finally, the training dataset consisted of 3212 images for the invasion-positive group and 4972 images for the negative group.

In the test dataset, we used all images of each case in both the invasion-positive and -negative groups. The invasion-positive group consisted of 86 images (69 invasion-positive slices and 17 invasion-negative slices; every case contained at least one invasion-positive slice) from 15 cases (non-contrast only: *n* = 1, contrast only: *n* = 10, both non-contrast and contrast: *n* = 2). The invasion-negative group consisted of 99 images from 22 cases (non-contrast only: *n* = 1, contrast only: *n* = 11, both non-contrast and contrast: *n* = 5). No data augmentation was performed on the test dataset.

We used transfer learning from a pre-trained CNN algorithm for image classification to distinguish the invasion-positive or -negative status of the axial CT images. The Residual Network 50 (ResNet50) was used as the original model in this work. The ResNet extracts residual features as a subtraction of features learned from the input of that layer using “skip connections.” The ResNet50 architecture contained one 3 × 3 convolutional layer, one max-pooling layer, and 16 residual blocks. Each block contained one 1 × 1 convolutional layer, one 3 × 3 convolutional layer, and one 1 × 1 convolutional layer. The residual connection was from the beginning of the block to the end of the block. The output of the last block was connected to a fully connected layer with a sigmoid function to make the prediction [16]. To train the model, while training the final fully connected layer, the previous layers’ parameters were kept at the original weights of the ResNet50. This enabled us to retain the broader features of the ResNet50 model and adapt the model to the CT images with a limited number of trainable parameters.

For the training session, we used the adaptive moment (Adam) optimizer. Hyperparameters were set to 15 epochs, a mini-batch size of 64, and the learning rate 1.0 × 10^−4^. During training, 30% of the training data was used for internal validation in transfer learning. The ResNet50 model can analyze the input image and generate a probability indicating the category to which it belonged. In this study, the model produced a binary classification of invasion-positive or -negative status for the test dataset.

**Fig. 2:**
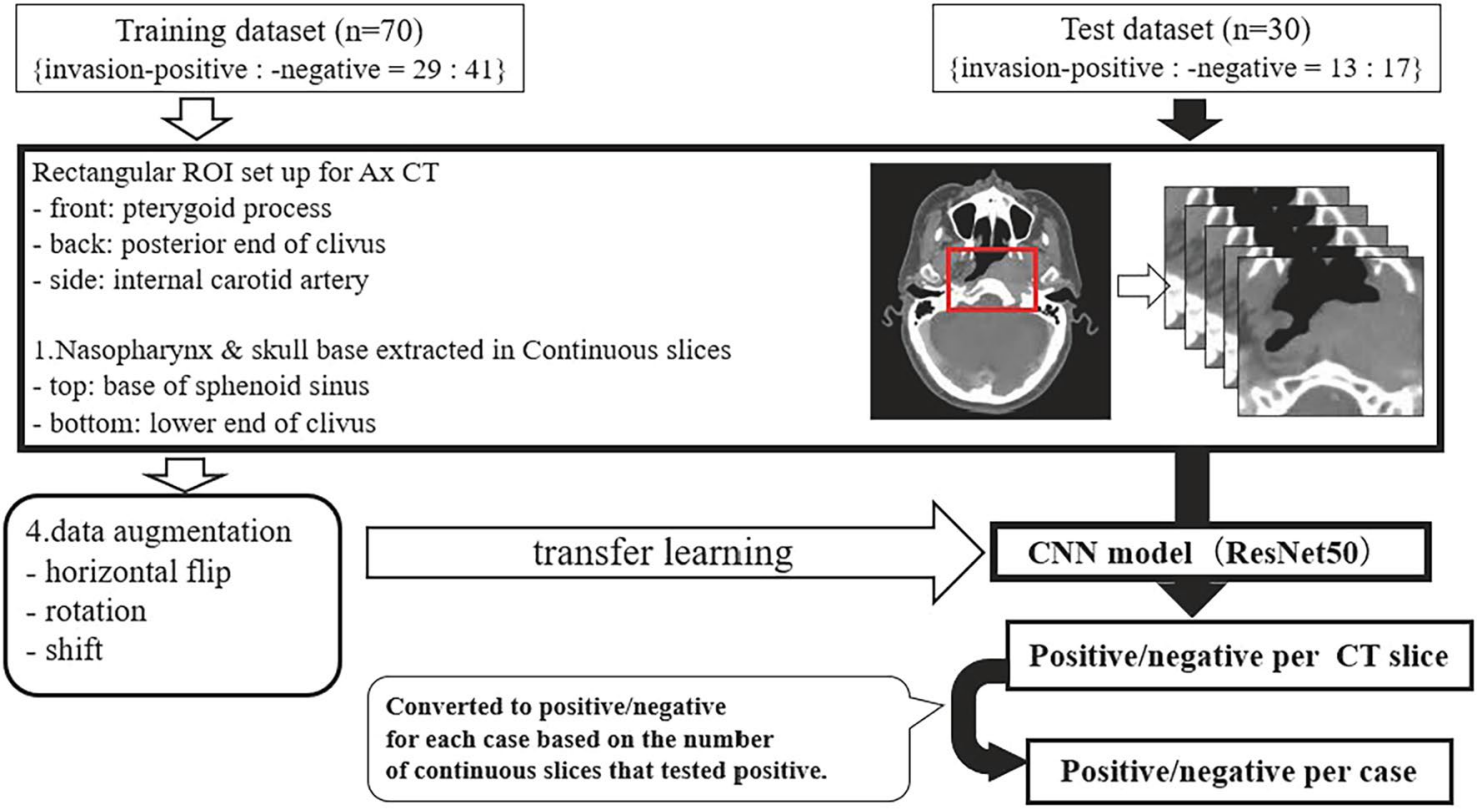
Image preprocessing on CT images for the deep-learning (DL) analysis. First, as image preprocessing, a rectangular region of interest (ROI) was manually placed to include the nasopharynx and skull base on axial CT images (*red rectangle*). The landmarks for the area of the ROI were the pterygoid process in the front, the posterior end of the oblique plateau in the back, and the internal carotid artery at the side. Next, images segmented by ROI were extracted as continuous slices in the range from the base of the sphenoid sinus to the lower end of the clivus. Finally, for the training dataset, data augmentation was performed by horizontally flipping, rotating, and/or shifting the images. The training dataset images, having undergone preprocessing and data augmentation, were trained on ResNet50 to create a diagnostic CNN model. This CNN model evaluated the preprocessed images of the test dataset, determining the positive/negative status for each slice. Based on the consecutive number of slices classified as positive by the CNN model, positive or negative determinations were made for each case

When evaluating the developed CNN model on the test dataset, the first step was to classify all slices as invasion-positive or -negative in each patient using the CNN model. The number of continuous slices that the CNN model determined to be invasion-positive was then added up and converted into a diagnosis for each patient. The image processing and deep-learning analysis steps are illustrated in Fig. 2.

The CNN model was established using an Ubuntu 18.04 long-term support-based server with a Core i9 10980XE 18-core/36-thread 3.0-GHz central processing unit, four NVIDIA Quadro RTX8000 graphics processing unit cards, and 128-GB (16 GB × 8) DDR4-2933 quad-channel memory for training and validation. Transfer learning took approximately 50 min. MATLAB (R2021a, MathWorks, Natick, MA, USA) and Metavol software (https://www.metavol.org) [17] were used for all image analyses.

### Visual evaluation by radiologists

Two radiologists, one of whom had extensive experience with head and neck imaging (senior reader (SR), 13 years’ radiology experience and board-certified) and the other with less experience (junior reader (JR), 1 year of radiology experience) reviewed the CT images of the test dataset and determined whether the nasopharyngeal tumor was invasion-positive or -negative from the base of the sphenoid sinus to the lower end of the clivus. All of the axial CT slices with a complete field of view (not segmented images) were used for evaluation, with appropriate adjustment of the window level/width for the evaluation.

### Visual representation with a Grad-CAM

Advancements in DL have facilitated the analysis of complex medical images with minimal human intervention. However, most DL models are often considered ‘black boxes’ due to their nonlinear underlying structures. This has led to the emergence of explainable artificial intelligence (XAI) research, which aims to enhance transparency in AI models and interpret the fidelity of their inferences [18]. Gradient-weighted class activation mapping (Grad-CAM) is one of the most commonly used XAI methods for enhancing the visual evaluation of the site at which a CNN model is focusing its attention during inference [19]. In the present study, the Grad-CAM was created by extracting feature maps from the convolutional layers, and it provided visualization with a heat map indicating where the attention of the network is highlighted according to the input images. We tested whether the CNN model was able to focus on the nasopharyngeal lesion itself and the clivus using the Grad-CAM heatmap on preprocessed CT images of the test dataset.

### ROI setting test by another radiologist

To evaluate the diagnostic results of the CNN model due to the differences in the person who set the rectangular ROI, another radiologist (2 years’ radiology experience) set the ROI on the axial-section CT images of the test dataset after teaching the extent of lesion extraction (see the ‘Image preprocessing’ section above).

### Statistical analyses

The diagnostic performances of the test dataset obtained with the developed CNN diagnostic model and that of the test dataset obtained by the two radiologists (SR and JR) were respectively evaluated. In the analysis of the CNN model, the optimal number of continuous invasion-positive slices to diagnosis invasion-positive or -negative status for each patient was determined from the Youden index using in the receiver operating characteristic (ROC) curve analysis. We evaluated the diagnostic performance by calculating the area under the ROC curve (AUC), accuracy, sensitivity, specificity, positive predictive value (PPV), and negative predictive value (NPV). The diagnostic performance of the CNN model alone was compared with the visual evaluation by each of the two radiologists. The χ^2^-test was used for the AUC comparisons. Statistical significance was defined as *p*-values < 0.05. All statistical analyses were performed using BellCurve for Excel (Social Survey Research Information Co., Tokyo).

## Results

### Patient characteristics

The characteristics of the 100 patients were as follows: the median (range) age was 60 years (18–85) and the male-to-female ratio was 78:22. Among the 94 cases of nasopharyngeal carcinoma, 89 were classified as squamous cell carcinoma (including low to undifferentiated types), and the other five cases were classified as other histological types such as adenocarcinoma. There were six cases of malignant lymphoma. The T-classification of nasopharyngeal carcinoma based on the patients’ medical records was as follows: T1 (*n* = 26), T2 (*n* = 18), T3 (*n* = 27), and T4 (*n* = 22); one case had no recorded T-classification.

### Diagnostic performance of the CNN model

For each patient in the test dataset, ROC curve analysis was performed to classify invasion-positive or -negative lesion using the number of continuous slices determined as invasion-positive by our newly developed CNN model based on ResNet50. The AUC of the CNN model was 0.953 (95% confidence interval [CI] 0.861–1.045). On the CNN model when two or more continuous slices were set as the best cut-off point based on the Youden index, the following values were obtained: 0.973 accuracy (95%CI 0.858–0.999), 0.933 sensitivity (95%CI 0.681–0.998), 1.000 specificity (95%CI 1.000–1.000), 1.000 PPV (95%CI 1.000–1.000), and 0.957 NPV (95%CI 0.781–0.999).

### Radiologists' diagnostic performances

The diagnostic performances of the two radiologists in evaluating the presence or absence of skull-base invasion on the test dataset CT images were as follows: SR: AUC 0.832 (95%CI 0.704–0.960), 0.833 accuracy (95%CI 0.653–0.944), 0.769 sensitivity (95%CI 0.462–0.950), 0.882 specificity (95%CI 0.636–0.985), 0.833 PPV (95%CI 0.516–0.979), and 0.833 NPV (95%CI 0.586–0.964). The corresponding values for the JR were: AUC 0.617 (95%CI 0.459–0.774), 0.600 accuracy (95%CI 0.406–0.773), 0.692 sensitivity (95%CI 0.386–0.909), 0.529 specificity (95%CI 0.278–0.770), 0.529 PPV (95%CI 0.278–0.770), and 0.692 NPV (95%CI 0.386–0.909).

**Fig. 3:**
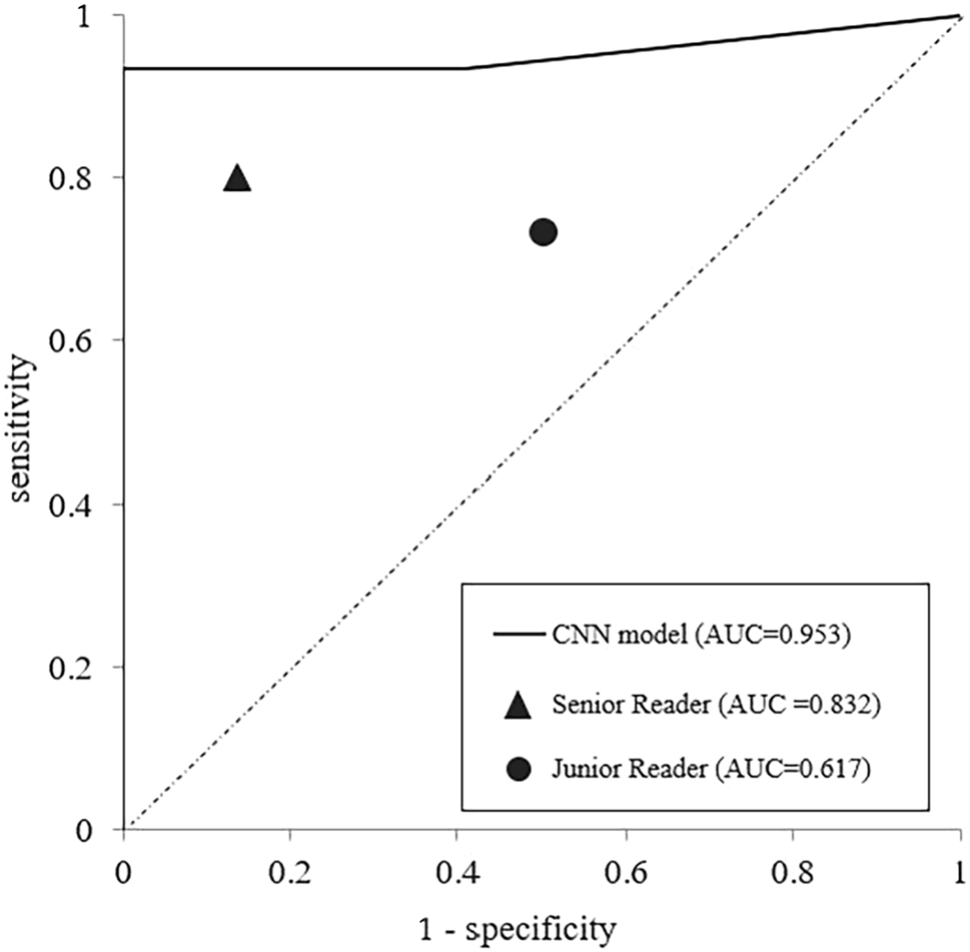
The ROC curve analyses by the CNN model and by the two radiologists. The ROC curve for the CNN-based deep-learning model of ResNet50 is shown. The point plots of the sensitivity and specificity values obtained by the two specialist radiologists (senior reader and junior reader) are also shown

Figure 3 shows the ROC curve obtained from the CNN model on the test dataset and the point plot of the sensitivity and specificity values obtained from the visual evaluation of the two radiologists. The diagnostic performance of the CNN model and that of the two radiologists are depicted in Table 1. The AUC was largest for the CNN model, the SR, and the JR, in that order, and there were also significant differences between these values (the CNN model vs. the SR: *p* = 0.050, the CNN model vs. the JR: *p* < 0.001, the SR vs. the JR: *p* = 0.012).

**Table 1:** The diagnostic performances

	CNN model	Senior Reader	Junior Reader
AUC	0.953	0.832	0.617
Accuracy	0.973	0.833	0.600
Sensitivity	0.933	0.769	0.692
Specificity	1.000	0.882	0.529
PPV	1.000	0.833	0.529
NPV	0.957	0.833	0.692

*AUC* area under the curve, *CNN* convolutional neural network, *NPV* negative predictive value, *PPV* positive predictive value

### Visual representation with Grad-CAM

**Fig. 4:**
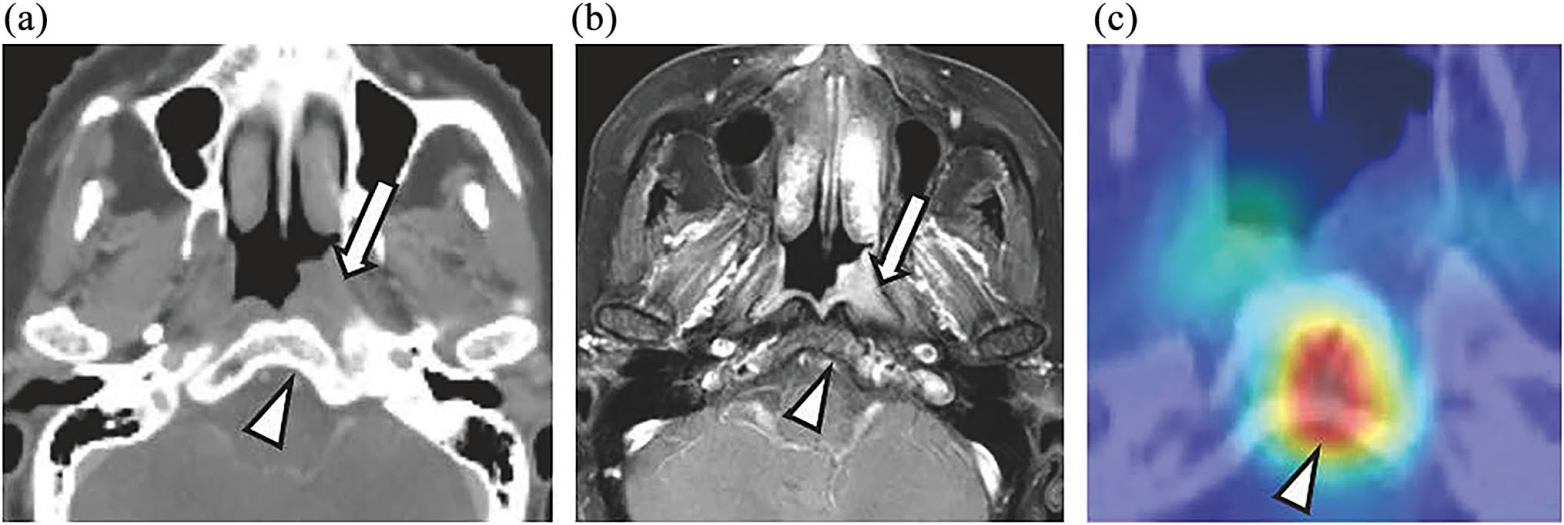
Representative case with a Grad-CAM heatmap: invasion-negative. **a** CT. **b** FsGdT1WI. **c** Grad-CAM heatmap. Nasopharyngeal carcinoma is seen on the left lateral wall (*white arrow*); the CT images show no bone destruction in the clivus, whereas a slight osteosclerotic change within the bone marrow was observed (*white arrowhead*); However, the MRI images show normal anterior vertebral muscles between the tumor and clivus and no remarkable contrast enhancement on the clivus (*white arrowhead*). The skull-base invasion classification is thus negative. The heatmap focused mainly on bone marrow without soft tissue density or osteosclerosis (*white arrowhead*)

**Fig. 5:**
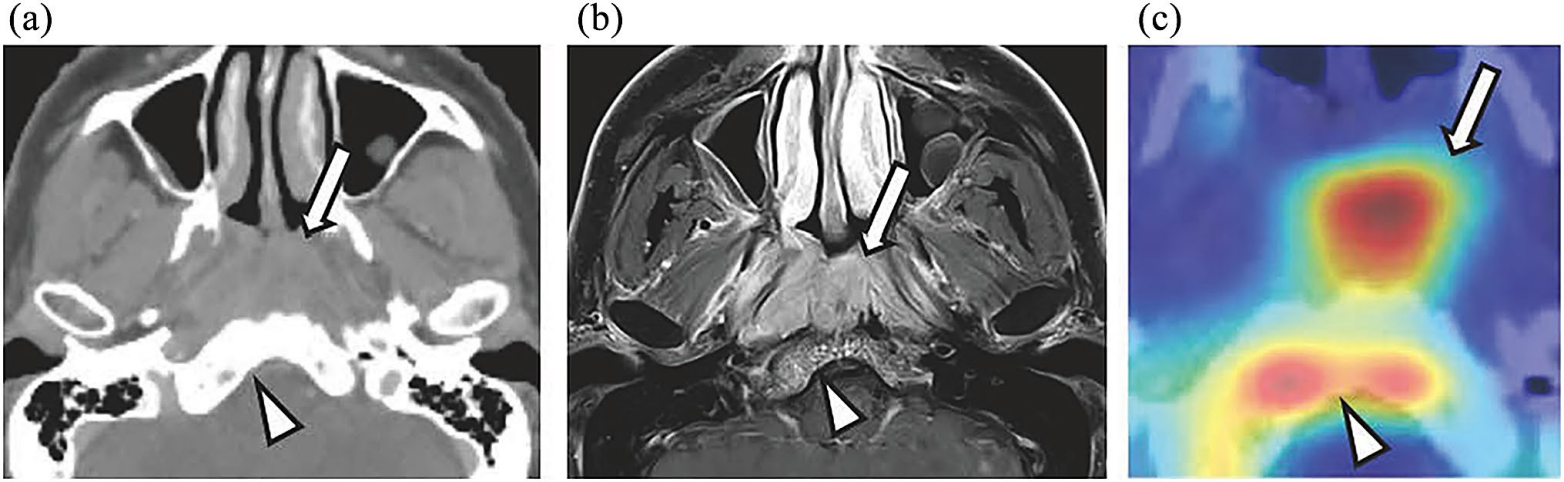
Representative case with a Grad-CAM heatmap: invasion-positive; Case 1. **a** CT. **b** FsGdT1WI. **c** Grad-CAM heatmap. Nasopharyngeal carcinoma occupies the pharyngeal cavity (*white arrow*), and the CT images show an osteosclerosis of the bone marrow, corresponding to the contrast enhancement on MRI images (*white arrowhead*). This indicates a finding of skull-base invasion. The heatmap focuses on the nasopharyngeal mass (*white arrow*) and the osteosclerosis of the bone marrow (*white arrowhead*). The CT images also show a possible erosion on the front of the clivus, but this area is located at the border of the hotspot on the heatmap; it was unclear whether the CNN model emphasized this finding

**Fig. 6:**
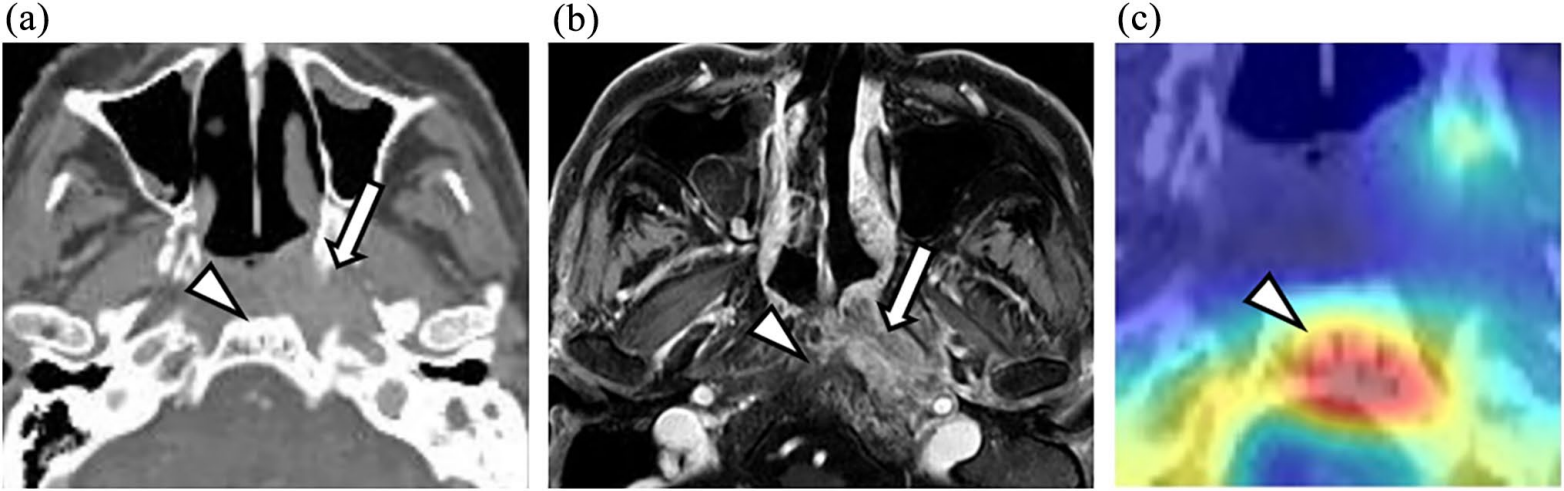
Representative case with a Grad-CAM heatmap: invasion-positive; Case 2. **a** CT. **b** FsGdT1WI. **c** Grad-CAM heatmap. Nasopharyngeal carcinoma is present on the left lateral wall (white arrow); CT images show faint osteosclerosis of the clivus as a slight high density area (white arrowhead). However, there is clear contrast enhancement on MRI images (white arrowheads). This indicates a finding of skull-base invasion. The heat map focuses on bone marrow osteosclerosis (white arrowheads). Notably, the CNN model successfully judged this case as invasion-positive, whereas the senior radiologist gave an incorrect diagnosis (i.e., invasion-negative)

Figures 4, 5, and 6 provide CT and MRI images of representative invasion-negative and positive cases combined with Grad-CAM heatmaps. The bright red areas of the Grad-CAM heatmaps indicate the areas where the CNN model is considered by the model to be most relevant for assessing the presence or absence of skull-base invasion by the tumor, followed by the heatmaps’ yellow and green areas, in sequence. In the invasion-negative cases, the heatmaps focused primarily on bone marrow of the clivus without osteosclerosis and/or soft tissue density, and a similar pattern was seen in all 17 invasion-negative cases with varying degrees of attention. In the invasion-positive cases, the heatmaps focused mainly on nasopharyngeal masses and/or osteosclerosis or bone destruction of the clivus, which was confirmed in 12 of the 13 invasion-positive cases (one false-negative case occurred).

### ROI setting test by another radiologist

**Fig. 7:**
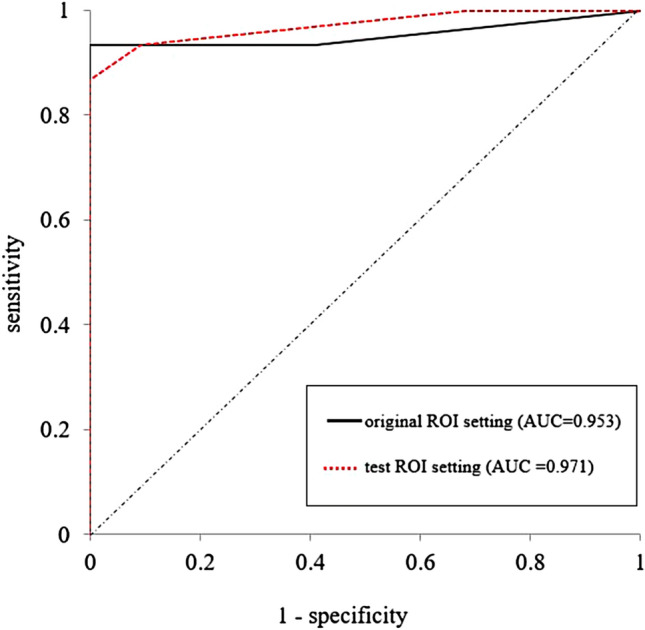
ROC curve analysis at the original and newly established ROI setting. The ROC curves obtained by the CNN model analysis at the original ROI setting in the test dataset (*black line*; the same curve provided in Fig. 3) and at the newly established ROI by another radiologist (*red line*) was respectively presented

The images extracted in the newly established ROIs in the test dataset were fed into the CNN model, and the ROC curve analysis of the invasion-positive or -negative classification for each patient was performed using the number of continuous invasion-positive slices (see the ‘Deep learning analysis’ section above). This AUC was 0.971 (95%CI 0.861–1.045), which is not significantly different from the ROC-AUC at the original ROI setting (*p* = 0.730) (Fig. 7).

## Discussion

Our results demonstrate that the use of the CNN technique for the diagnosis of skull-base invasion by nasopharyngeal malignant tumors in CT images was successful. The trained radiologists were more accurate in diagnosing skull-base invasion than the untrained radiologist, and the developed diagnostic model showed higher diagnostic performance than either type of radiologist. Deep learning techniques thus have the potential to provide high diagnostic accuracy in investigations of skull-base invasion on CT images, even without the contribution of an experienced radiologist. With the further development of this technique, it is expected that accurate staging of nasopharyngeal malignant tumors and appropriate treatment selection will be possible, ultimately leading to improved patient prognosis.

Although CT is an effective modality for evaluating bone, especially cortical bone invasion, CT is subject to beam-hardening artifacts and limited resolution of contrast enhancement in the bone marrow, which can make it difficult to assess bone invasion [20]. The diagnostic performance of different modalities for skull-base invasion in nasopharyngeal carcinoma has been described [20, 21], and in one of those studies, conventional CT had 78.6% sensitivity and 86.1% specificity, while combining bone subtraction iodine imaging using area-detector CT achieved 92.9% sensitivity and 95.6% specificity [20]. The other study was validated with dual-energy CT and used simulated single-energy CT; the study’s authors reported 75.0% sensitivity and 93.2% specificity, and when the iodine concentration and the effective atomic number (Zeff) values were combined, 90.7% sensitivity and 95.3% specificity were observed [21].

However, these imaging devices or methods require new technology, and the number of facilities where such imaging is available is limited. We find that the diagnostic performance of the developed CNN model (93.3% sensitivity and 100% specificity) is comparable to the results of the above-cited studies. Our present findings were also obtained with only conventional CT images, which unlike the above-mentioned studies, may be available at any facility. Although we did not use a fully automated pipeline in this study (since manual setting of the rectangular ROIs was required), almost the same diagnostic performance was successfully obtained when another junior radiologist conducted the ROI placement. This result indicates that the interobserver variability will be acceptable for clinical use.

Investigations of the usefulness of deep-learning approaches to diagnose the presence of local invasion by malignant tumors in CT images have included examinations of visceral pleural invasion in early-stage lung cancer [12], muscular invasion in bladder cancer [13], and extranodal extension in lymph node metastases of head and neck squamous cell carcinoma [14]. However, to the best of our knowledge, there are very few similar reports on head and neck tumors, with the most recent being the 2022 application of deep-learning approache to the diagnosis of orbital invasion by nasal/sinonasal tumors [15]. The applications of CNN techniques for nasopharyngeal tumors (mainly carcinoma) have gradually increased in recent years, including image segmentation [22, 23], disease classification [24], prognosis prediction [25], and the prediction of treatment response [26]. However, most of these studies included MRI findings as training data for use, and few used CT results as training data. In addition, to the best of our knowledge, there are no reports of CNN models for diagnosing skull-base invasion by nasopharyngeal tumors, which we consider novel results of the present study.

A Grad-CAM has been demonstrated to be able to provide a visual and intuitive understanding of the rationale behind CNN classification. For example, in a study that classified subsolid nodules in the lungs into three classes (benign/preinvasive lesions, minimally invasive adenocarcinomas, and invasive adenocarcinomas), the Grad-CAM heatmaps focused on the shape of the lesion’s edges, consolidation components, and air bronchograms, which aligned with the important features that radiologists should evaluate when diagnosing malignant nodules [27]. In our present investigation, the Grad-CAM heatmaps focused on the normal bone marrow of the clivus without ossification and/or the soft tissue density in the invasion-negative cases. In contrast, the heatmaps focused on the nasopharyngeal tumor itself and/or the ossification or bone destruction of the clivus in most of the invasion-positive cases. These findings might be key points to which radiologists pay attention when evaluating the presence or absence of skull-base invasion in daily clinical practice.

This study has some limitations. The sample size was small (*n* = 100) due to the single-institutional study design, and the results should thus be treated as preliminary. We partly overcame the sample size limitations by data augmentation. In addition, due to the standard practice of chemoradiotherapy for the treatment of nasopharyngeal cancer, there was a lack of histopathologic verification of skull-base invasion. In reports regarding the laryngeal cartilage, in areas adjacent to the tumor, contrast enhancement can also be caused by reactive inflammation, edema, and fibrosis [28], raising the possibility of false positives in the present patients’ MRI findings. However, it is important to note that the treatment decisions were based primarily on the presence of signal changes indicative of invasion, and there was no significant discrepancy between the clinical judgments and the ground truth in this study. Finally, CNN model training was performed using only axial CT images with specific window level/width settings. Visual evaluation by both radiologists (JR and SR) was also performed using axial CT only, although window level/width adjustment was available. These settings were somewhat divergent from the actual clinical situation.

## Conclusions

The CNN-based deep learning technique can be valuable for diagnosing skull-base invasion by nasopharyngeal tumors on CT images. This technique may become a useful diagnostic support tool, especially in medical institutions and radiology departments in which there is a lack of expertise in head and neck imaging.

## Abbreviations

Adam: Adaptive moment
AUC: Area under the curve
CNN: Convolutional neural network
CT: X-ray computed tomography
DL: Deep learning
DICOM: Digital imaging and communication in medicine
FsGdT1WI: Fat-suppressed gadolinium-enhanced T1-weighted imaging
FsT2WI: Fat-suppressed T2-weighted imaging
Grad-CAM: Gradient-weighted class activation map
GdT1WI: Gadolinium-enhanced T1-weighted imaging
HU: Hounsfield units
JPEG: Joint photographic experts group
JR: Junior reader
MRI: Magnetic resonance imaging
NPV: Negative predictive value
PPV: Positive predictive value
ResNet: Residual network
ROC: Receiver operating characteristic
ROI: Region of interest
SR: Senior reader
T1WI: T1-weighted imaging
XAI: Explainable artificial intelligence
